# Initial experience with Duplex scan combined with contrast-enhanced ultrasound for follow-up of endovascular abdominal aortic aneurysm repair

**DOI:** 10.1590/1677-5449.200093

**Published:** 2021-11-01

**Authors:** Carolina Brito Faustino, Carlos Ventura, Maria Fernanda Cassino Portugal, André Brunheroto, Marcelo Passos Teivelis, Nelson Wolosker

**Affiliations:** 1 Hospital Israelita Albert Einstein – HIAE, São Paulo, SP, Brasil.; 2 Faculdade de Medicina da Universidade de São Paulo – FMUSP, São Paulo, SP, Brasil.

**Keywords:** microbubbles, EVAR, endoleaks, ultrasound

## Abstract

**Background:**

Microbubble contrast enhanced ultrasound (CEUS) is an accurate diagnostic method for follow-up after endovascular abdominal aortic aneurysm repair (EVAR) that has been well-established in international studies. However, there are no Brazilian studies that focus on this follow-up method.

**Objectives:**

The objective of this study was to report initial experience with CEUS at a tertiary hospital, comparing the findings of CEUS with those of conventional Doppler ultrasound (DUS), with the aim of determining whether addition of contrast to the standard ultrasonographic control protocol resulted in different findings.

**Methods:**

From 2015 to 2017, 21 patients in follow-up after EVAR underwent DUS followed by CEUS. The findings of these examinations were analyzed in terms of identification of complications and their capacity to identify the origin of endoleaks.

**Results:**

There was evidence of complications in 10 of the 21 cases examined: seven patients exhibited endoleaks (33.3%); two patients exhibited stenosis of a branch of the endograft (9.52%); and one patient exhibited a dissection involving the external iliac artery (4.76%). In the 21 patients assessed, combined use of both methods identified 10 cases of post-EVAR complications. In six of the seven cases of endoleaks (85.71%), use of the methods in combination was capable of identifying the origin of endoleakage. DUS alone failed to identify endoleaks in two cases (28.5%) and identified doubtful findings in another two cases (28.5%), in which diagnostic definition was achieved after employing CEUS.

**Conclusions:**

CEUS is a technique that is easy to perform and provides additional support for follow-up of infrarenal EVAR.

## INTRODUCTION

After endovascular repair of an abdominal aortic aneurysm (EVAR), patients need complete follow-up for the rest of their lives, for surveillance of possible complications related to the procedure, such as persistent aneurysm expansion, de novo aneurysm formation, potential rupture, internal leaks, and others.^1^ In general, follow-ups are recommended at 1, 6, and 12 months and then annually thereafter.^2^ Computed tomography angiography (CTA) is currently the imaging method of choice for postoperative follow-up.^2^^,^^3^ However, there are concerns with regard to the expense involved and the risks of collateral clinical effects.^4^^-^6

Doppler ultrasonography (DUS) is a noninvasive, accurate, and less expensive alternative. However, it is subject to certain limitations that are inherent to the assessment method, such as with patients who are obese, with gaseous abdominal distension, or with extensive atheromatosis of the artery wall. It is also an operator-dependent method in which inter and intra-operator variability can compromise consistent results.^7^ Combining additional technological tools such as image fusion and volumetric techniques with DUS increases its sensitivity and specificity,^8^^-^10 yielding greater diagnostic accuracy for possible complications, to the point at which some authors now suggest its use as a less invasive option to substitute angiotomography for follow-up of EVAR patients.^11^

Use of contrast ultrasonography (CEUS) has emerged as an additional technique^12^^,^^13^ and has been proposed in international studies as an accurate method for screening for complications after EVAR, with specific indications for checking for endoleaks and, in other cases, studying the morphology of atherosclerotic plaques.^1^^,^^7^^,^^14^^,^^15^ However, no studies have been conducted in Brazil to investigate this follow-up method in our country.

The objective of this study was to report the initial experience with CEUS at a tertiary hospital in the city of São Paulo, SP, Brazil, comparing the findings of methods performed with and without contrast to test whether addition of contrast to the standard DUS protocol changes the findings in any way.

## METHODS

A total of 21 patients who were in postoperative follow-up after EVAR were recruited from 2015 to 2017 at the Hospital Israelita Albert Einstein, São Paulo, SP, Brazil. All patients were males, aged from 65 to 83 years (mean 80 years, median 73 years), for whom DUS had been ordered as a follow-up method.

The study was approved by the institutional Human Research Ethics Committee, under protocol 06201218.1.0000.0071 (decision number 3.745.321).

In eight of these patients, ultrasonography had been ordered because CTA had to be substituted with another method due to a contraindication (borderline renal function). In one case, it was requested as a supplementary examination to attempt to understand CTA findings suggestive of endoleaks that were inconclusive. In the remaining 12 cases, ultrasonographic examination was requested as the first choice at that point in follow-up.

**Figure 1:**
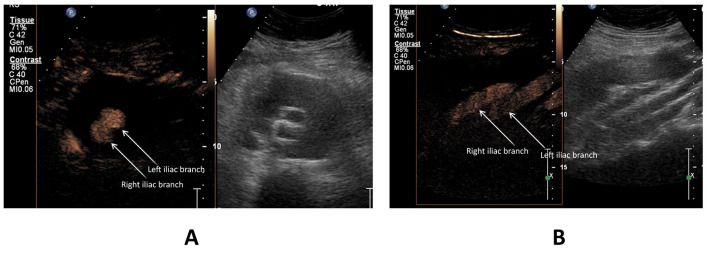
Aneurysm sac of the abdominal aorta with bifurcated endograft, with no abnormal findings. (A) Transverse plane; (B) longitudinal plane. Both images show that both legs of the endograft are patent and have customary morphology (arrowed), with no evidence of leaks. Figures A and B show a feature of the ultrasound machine that presents split images (side by side); in each case the left half of the figure shows the image with the contrast preset (pulse-inversion harmonics) and the right half shows the B-mode image for comparison.

Patients were examined with the conventional DUS technique and then the technique was performed with the addition of microbubble contrast (CEUS) (Figure 1). Findings observed using a combination of both methods were considered the gold standard for comparison between DUS only vs. DUS combined with CEUS.

At our institution, addition of contrast to the DUS examination increased the cost by 30.4% (R$ 516.03 per abdominal aorta examination).

### Examination technique

Examinations were preferably conducted in the morning, after a 6-hour fast. The ultrasound machines used were EPIQ and IU 22 models (Philips, Montana, United States) coupled to a convex transducer (from 1-5 MHz to 2-5 MHz), with optimized processing parameters.

All examinations were conducted and reported by the same examiner. Patients were examined in the supine position and if necessary in left or right lateral recumbent positions, with initial assessment in B-mode, followed by addition of color mode, and then by use of contrast with an appropriate preset, and ending by reassessing in color mode to check findings, according to the protocol described below.

### Examination in B-mode

The B-mode examination includes longitudinal and transverse examination of the aorta and aneurysm sac, The primary objectives are to examine aorta morphology, the position and integrity of the endograft, and the dimensions and echogenicity of the aneurysm sac (anechoic areas were considered suggestive of leakage).

### Examination in color mode

The ultrasound machine’s signal processing parameters were set to maximum gain and the smallest scale possible with adequate image formation, with the intention of achieving the maximum possible flow detection sensitivity to confirm leaks.

### Examination with contrast (CEUS)

Sonovue contrast was employed. This is sold in a kit containing sulfur hexafluoride powder and a syringe containing solvent (5 mL). It should be handled gently during preparation and injection to avoid rupturing the microbubbles.

A 1.0 mL contrast bolus followed by a 10 mL saline solution flush is administered to the patient by slow intravenous injection, preferably via an antecubital vein, with an 18 G needle. The ultrasound machine’s specific preset for contrast was used, set for pulse-inversion harmonic imaging with a low mechanical index (0.4). Another important consideration for use of this contrast is to remove the scanner’s focal zone from the area of interest to avoid premature rupture of the microbubbles. The contrast injection procedure was repeated up to three times as needed.

### Completion of the examination

After the examination with contrast, the color Doppler examination was repeated to confirm findings. It is important to note that the color Doppler gain should be adjusted to avoid blooming artifacts caused by residual contrast.

Analysis of the dynamic behavior of the microbubbles was performed using both static and continuous images, during the several different phases of contrast circulation. Dynamic image sequences were recorded in video mode using the internal buffer of a digital display for later analysis.

The timer was started at the start of contrast injection, most importantly to analyze occurrences of highlighting/leakage that occur later and, consequently, are less intense.

### Data analysis and statistical assessment

The indications for ultrasound examinations, occurrence of complications, and capacity to diagnose lesions were analyzed. Specifically in cases in which endoleaks occurred, the difference in methods’ capacity to detect the origin of leakage was also analyzed. The DUS and CEUS methods were compared in terms of their capacity to detect leakage into the interior of the aneurysm sac.

## RESULTS

In all cases, the examinations were ordered by the vascular surgeons responsible for the cases, with the objective of post-EVAR follow-up and assessment of possible complications. There were no complications related to the examinations, regardless of method.

Post-EVAR complications were diagnosed in ten of the 21 patients assessed (47.62%) (Table 1). Four of the endoleak cases (33.33%) were classified with CEUS as type II, two as type Ib, and one as type III.

**Table 1:** Complications identified after EVAR in a group of patients examined with both methods.

	**Identified with DUS alone, n (%)**	**Identified with DUS and CEUS, n (%)**
Endoleaks*	3 (14.28)†	7 (33.33)
Type II	4 (57.14)	4 (57.14)
Type Ib	2 (28.57)	2 (28.57)
Type III	0	1 (14.28)
Adequate identification of origin	6 (85.71)‡	7 (100)
Stenosis of endograft branch	2 (9.52)	2 (9.52)
Iliac dissection	1 (4.76)	1 (4.76)

* All endoleaks identified were type II;

† In two cases, DUS identified changes suggestive of endoleaks, but did not define a diagnosis;

‡ After determination that endoleaks were present and classification of their origin by CEUS, DUS alone was able to identify the origin of leakage in 6 out of 7 cases. EVAR = endovascular abdominal aortic aneurysm repair; DUS = duplex ultrasonography.

### Comparison of examination methods

**Figure 2:**
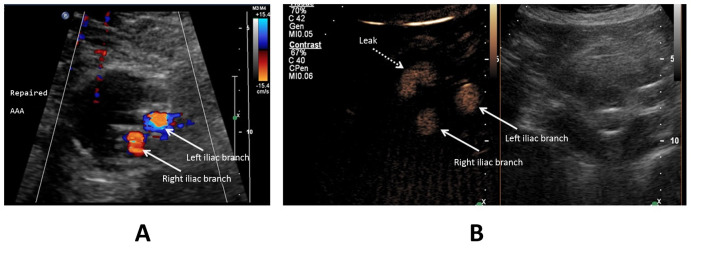
Aneurysm sac in an abdominal aorta with a bifurcated endograft and discrete late leakage. (A) Color Doppler ultrasonography in the transverse plane showing both legs of the endograft (arrowed) and no leakage; (B) Ultrasonography with contrast shows that there is discrete leakage to the left of the of the aneurysm sac (dotted arrow). This image is also split (side by side) using the feature described in Figure 1.

**Figure 3:**
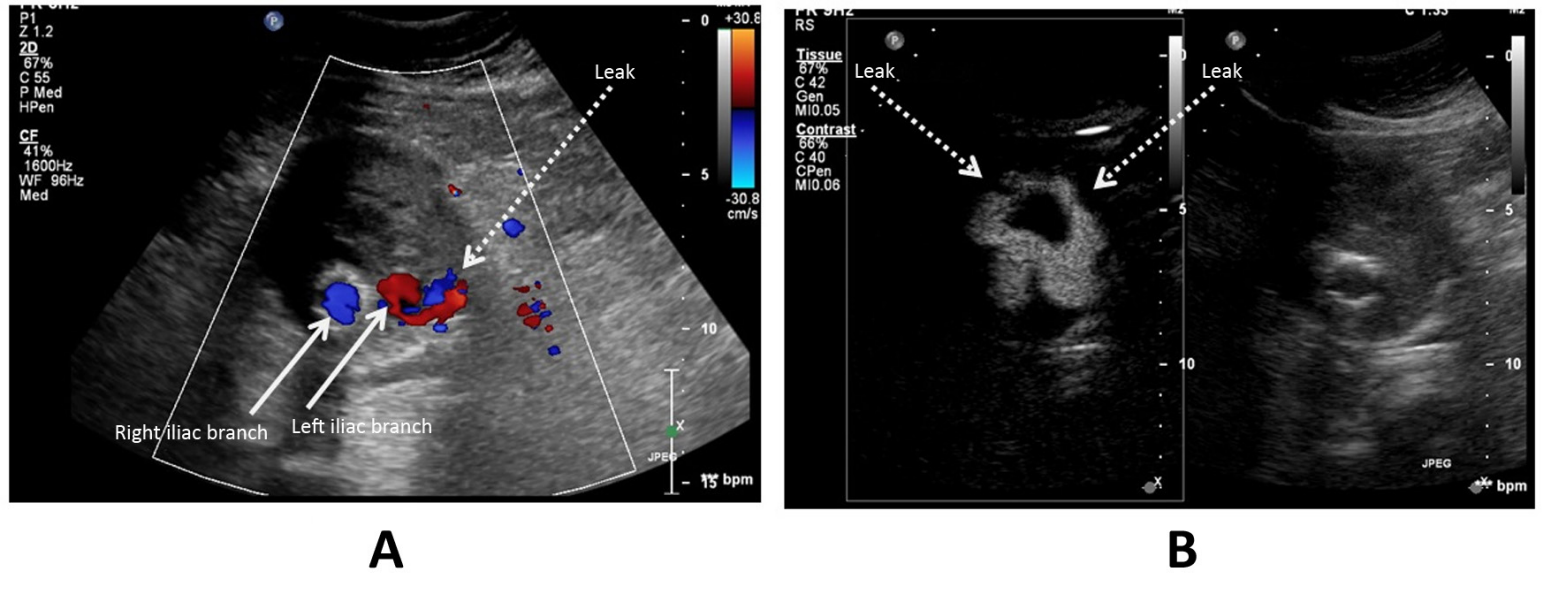
Aneurysm sac of an abdominal aorta with a bifurcated endograft and type IB leak. (A) Color Doppler ultrasonography in the transverse plane shows both legs of the endograft (arrowed) and a small leak at the distal portion of the left leg of the endograft (dotted arrow), which in the dynamic examination was confirmed as a type IB leak; (B) Ultrasonography with contrast shows that the extent of leakage is considerably larger than it appeared on the color Doppler scan (dotted arrows). Once more, the image is split (side by side) using the feature described in Figure 1.

**Figure 4:**
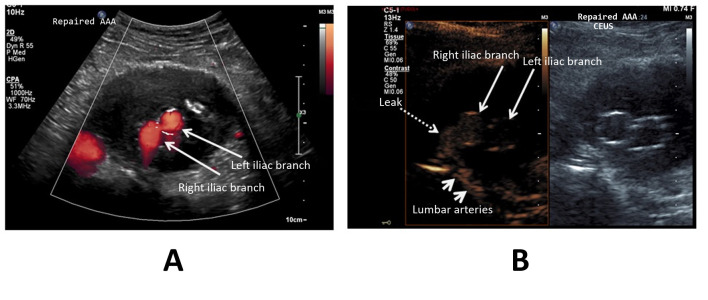
Aneurysm sac of an abdominal aorta with a bifurcated endograft and type II leak from the right lumbar artery. (A) Power Doppler ultrasonography in the transverse plane showing both legs of the endograft patent (arrowed) and no leaks; (B) Ultrasonography with contrast in the transverse plane is able to show leakage at the right posterior portion of the aneurysm sac (dotted arrow) originating from the lumbar artery (arrowheads). Once more, the image is split (side by side) using the feature described in Figure 1.

**Figure 5:**
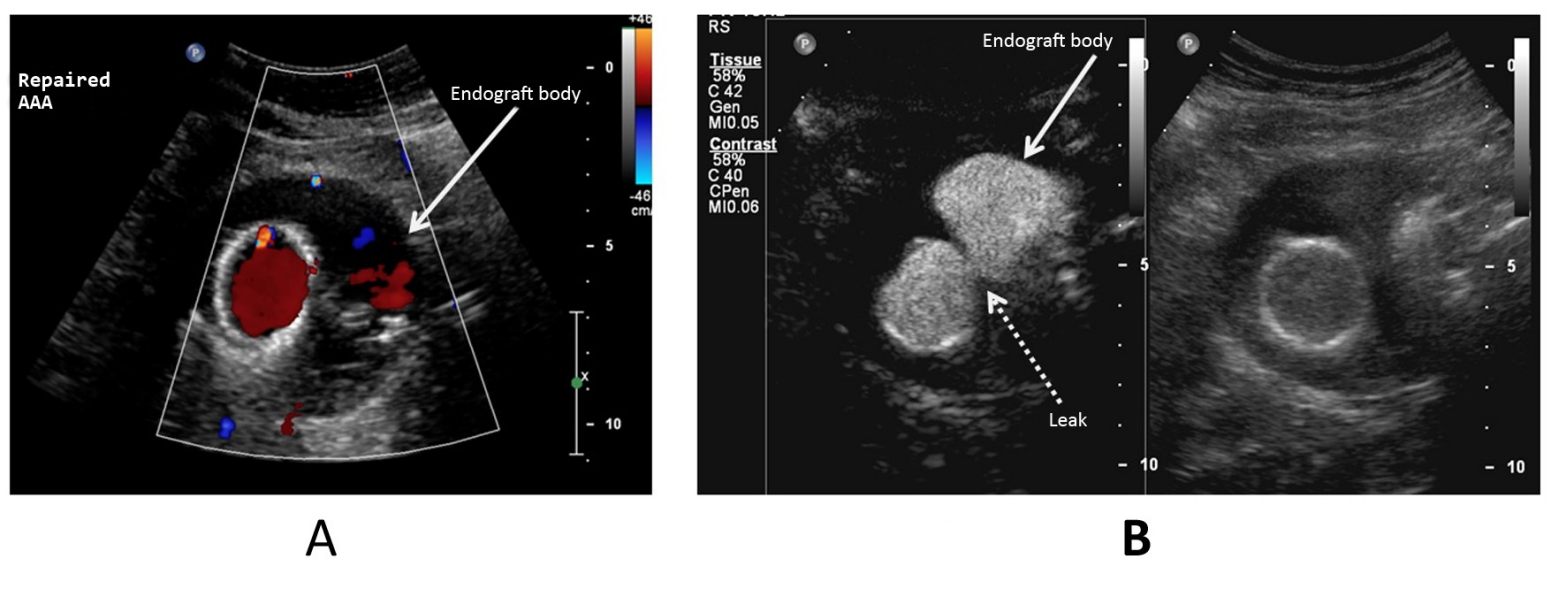
Aneurysm sac of abdominal aorta with a bifurcated endograft and type III leak. (A) Color Doppler ultrasonography in the transverse plane shows the endograft body on the left, patent and with continuous flow (arrowed); (B) Ultrasonography with contrast in the transverse plane shows that the leak is more significant (dotted arrow) and that the leak is a type III, in contact with the endograft body (dotted arrow). Once more, the image is split (side by side) using the feature described in Figure 1.

In regard to the capacity to detect leakage into the aneurysm sac interior, two of the seven cases of leakage that were identified by the end of the CEUS examination had been undetectable using DUS alone (28.5%) (Figure 2). In a further two cases (28.5%), DUS identified findings that could be considered leaks, but which were better characterized using CEUS, with confirmation of the endoleak diagnosis (Figures 3-5).

With regard to the capacity to detect the origin of the endoleaks, after determination of the existence of endoleaks and classification of their origins using CEUS, DUS alone was capable of identifying the origin of leakage in six out of seven cases (85.71%).

## DISCUSSION

Adding CEUS to the conventional Doppler examination enables structural vascular assessment of the macrocirculation (presence or absence of flow through the vessel and morphology of the lumen) and of the microcirculation and also evaluation of contrast uptake or tissue perfusion (characterization of the pattern and intensity of tissue uptake).^12^ The method with contrast provides detailed information of the blood supply to tissue lesions, similar to that provided by CTA and magnetic resonance angiography (MRA), since it offers hemodynamic information during the various different vascular phases; with the result that its initial applications were for assessment of hepatic nodules, for which it showed good accuracy,^16^^,^^17^ after which its applications were extended to many different areas.

Several studies have confirmed the method’s application for examination of the aorta and peripheral vessels, especially in relation to post-EVAR follow-up, to check for endoleaks and other complications.^7^^,^^11^^,^^14^^,^^18^^-^22 CEUS has also been studied for analysis of carotid diseases, notably for differential diagnosis between occlusion and pseudo-occlusion of the internal carotid artery and for characterization of microcirculation in unstable carotid plaques.^20^^,^^21^^,^^23^^,^^24^

Currently, the most widely-used ultrasonographic contrasts are fluorocarbon gases with high molecular weight. This confers lower solubility and diffusibility, resulting in more stable microbubbles, which remain in the circulation longer (up to 10 min), guaranteeing the method good sensitivity.^25^^-^27 Since they have diameters in the range of 1 to 10 μm,^19^ similar to red blood cells (7 μm), microbubbles mimic their behavior, enabling adequate assessment even in the microcirculation.^12^^,^^13^ Interaction with human albumin, phospholipids, surfactants, and other compounds^28^ acts to stabilize the microbubbles, reducing their diffusion, which not only contributes to the length of time they remain in circulation, but also increases the bubbles’ capacity to withstand pressure variations caused by the effects of the ultrasound and of cardiac contractions.^25^ Use of ultrasonographic contrast is contraindicated in patients with cardiac shunts, pulmonary hypertension, or unstable cardiopulmonary conditions (acute myocardial infarction, acute coronary syndromes, unstable congestive heart failure, severe ventricular arrhythmias, or respiratory insufficiency, including patients on mechanical ventilation), and also in patients with hypersensitivity to perfluorocarbon.^29^

In our sample, there were no cases of adverse reactions to the contrast. The most common adverse effects reported in the literature are pain or paresthesia at the puncture site, lumbar pain, and, rarely, allergic reactions, and damage to the microcirculation.^30^ Rare severe reactions have been reported, such as fatal cardiac or respiratory arrest, loss of consciousness, convulsions, symptomatic arrhythmia (atrial fibrillation, supraventricular tachycardia, ventricular fibrillation, or tachycardia), arterial hypotension, respiratory distress, or cardiac ischemia.^31^ It is recommended that patients be closely observed during and after administration of the contrast medium and to ensure presence of a cardiopulmonary resuscitation team and equipment.

A recent review of 18,942 consecutive echocardiography studies evidenced that the short-term (24 hours) mortality rates are similar for patients given a sonic contrast medium (Definity^®^, Bristol-Myers Squibb Medical Imaging Inc., New York, United States) during echocardiogram examinations performed at rest and patients who were not given contrast, with no difference in mortality.^32^ A multicenter prospective study published in 2008 in The American Journal of Cardiology,^13^ also detected no increase in mortality among patients who underwent echocardiography with contrast, when compared to patients examined without contrast. These results indicate that there is no additional risk associated with studies undertaken with CEUS.

In order to compare CEUS against other available imaging methods, such as digital subtraction angiography (DSA), CTA, MRA, and even arteriography, it is important to consider the potential of iodinated contrast and gadolinium to cause harm.^4^^,^^5^^,^^33^ Iodinated contrast, used in both SA and CTA, can cause immediate adverse reactions, which occur in 3.1% of patients when low osmolality contrast is used.^4^^,^^5^^,^^34^ Gadolinium chelate contrasts are used in magnetic resonance imaging and can also cause adverse reactions.^6^^,^^35^ The greater part of these reactions are mild and can be treated in radiology department.^4^ It should also be remembered that many patients with a history of reactions to iodinated contrast appear to be at increased risk of allergy to gadolinium chelate.^4^^,^^5^

In our study, we observed that adding contrast to the ultrasonographic EVAR follow-up protocol increased its capacity to detect endoleaks, compared with use of DUS only. In our sample, the combined method also increased the number of successful attempts to identify the origin of endoleaks. In agreement, a Cochrane Vascular Group review published in 2017^7^ analyzed the diagnostic accuracy of DUS and CEUS in terms of sensitivity and specificity for detection of internal leaks after EVAR, demonstrating that both USG methods (with or without contrast) exhibited high specificity. For diagnosis of leaks, however, the authors concluded that CEUS demonstrated superiority over DUS. They also concluded that CEUS could be included as a diagnostic method in endoleak surveillance programs, to be followed by CTA only when a positive ultrasound examination established the type of internal leakage and the treatment.^7^

Another systematic review of post-EVAR surveillance, conducted by Zaiem et al. and published in 2018 in the Journal of Vascular Surgery,^1^ assessed the ideal method and frequency of surveillance after EVAR in adult patients with abdominal aortic aneurysms. This review identified high rates of complications, particularly in the first year, and observed that MRA achieved the highest rate of detection of complications of the methods analyzed, followed by computed tomography (CT). In turn, DUS alone was less sensitive but more specific than CT for detection of endoleaks, whereas CEUS achieved high sensitivity. In this study, the results of the CEUS method for detection appeared to be equal to those of CT, with greater sensitivity for identification of late type II endoleaks. Both DUS alone and CEUS were highly specific for types I and III leaks and their estimated sensitivity tended to the same rates, although reliability was lower. The authors hypothesized that the highest rates of detection of leakage into the aneurysm sac were achieved with surveillance approaches employing combined tests; but the quality of evidence on diagnosis in this review was moderate, since it was derived from observational studies.^1^

To date, CTA remains the technique of choice for follow-up of complications after EVAR,^36^ because of its high sensitivity and specificity for assessing complications, especially endoleaks. Nevertheless, some authors suggest that ultrasonography may be more precise for classifying leaks,^15^ which is a fundamental step in defining management. CTA is capable of assessing leaks, even when flow is limited;^37^ however it does not provide information on the direction of blood flow and, because of this, is not always able to correctly determine the origin of endoleaks or classify them.^38^ In this context, ultrasonography can constitute a decisive option. Indeed, in our sample, the combination of CEUS and DUS achieved identification of the origin in 85.7% of endoleak cases.

In a 2003 study, Bendick et al.^14^ assessed 20 patients in post-EVAR follow-up using CTA. In their sample, eight endoleak cases were identified with CTA and were also identified with CEUS. In another two patients, CEUS identified proximal type endoleaks that were not diagnosed with CTA, but were confirmed with arteriography (the authors hypothesized that metallic artifacts probably compromised the ability to see the leaks with CTA). Additionally, in three cases in which it was not possible to classify endoleaks using CTA, the CEUS method correctly identified the type.^14^

Napoli et al.^39^ followed-up 112 patients who had undergone EVAR using CTA and assessed the role of CEUS in a group of patients diagnosed with aneurysm sac growth, in whom leakage was not identified with CTA or DUS. The patients were allocated to three groups: patients with aneurysm sac growth, in whom leakage was not identified with CTA or DUS (group A); patients without aneurysm sac growth and with no leakage diagnosed (group B); and patients in whom leaks were diagnosed and classified with CTA (group C). Patients in groups A and C also underwent arteriography for confirmation. CEUS correctly identified leakage in all patients in group A and classified the type of leak in 80% of them. Arteriography confirmed the result in 8 out of 10 patients. Arteriography was also unable to clear up doubts in cases in which classification of the type of endoleak was not possible with CEUS. In the control groups, CEUS did not produce any false positive or false negatives.^39^

In a study published in 2006,^40^ 10 patients with endoleaks diagnosed with CTA were examined with CEUS for classification or reclassification of leaks. CEUS confirmed the classification achieved with CTA in seven cases. In three cases endoleak classifications diverged between the methods: in all three conflicting cases arteriography confirmed the classification determined using CEUS. The authors of this study postulated that CEUS enables better evaluation of the origin of leakage because it shows flow in real time and is more specific than CTA for classification of endoleaks, providing more precise information for treatment planning.^40^

Although CTA remains an indispensable follow-up method and is still considered the gold standard during the postoperative period after EVAR, due to its ability to more precisely assess anchoring, integrity, and morphology of endografts,^3^ studies suggest that the CEUS method is safe and not inferior to CTA for diagnosis of complications and can be used for standard follow-up, with occasional supplementation with CTA when necessary.^7^ Some authors even propose that CEUS is more reliable than CTA for classification and assessment of leaks. The method is also indicated in cases in which the aneurysm sac grows, but no leakage is found with CTA, and for follow-up of type II endoleaks, with reductions in cost and in exposure to iodinated contrast and radiation.^40^

Although this and other studies have shown that addition of CEUS to the conventional ultrasonography protocol for EVAR follow-up constitutes a tool with the capacity to positively change the results of diagnostic investigation, at the time that the conclusions of this study were written, there was not enough evidence to support its routine use or its use as a substitute for other methods and no cost-benefit studies have been conducted in Brazil.

## LIMITATIONS

This is a study based on an institution’s initial experience of application of the contrasted ultrasonographic method, with a limited sample, in which the objective was limited to observing additional findings contributed by using contrast in comparison to the conventional DUS examination protocol for post-EVAR follow-up. The study was based on a heterogeneous sample, since it included cases in which there were contraindications to CTA, and also cases that had already undergone CTA with limitations.

The most important limitation of the study is that it did not make any comparison between the methods analyzed (DUS alone and DUS combined with CEUS) and angiotomography, which is still considered the gold standard for post-EVAR follow-up.^2^^,^^3^ Since the parameter employed was the findings of the combined method, the study is only of use for identification of the additional benefit yielded by adding scans with contrast medium to the conventional DUS examination, since it does not have a design that would enable validation of a diagnostic method.

Studies specifically designed for validation of diagnostic parameters are needed to define, in Brazil, the ideal application of the CEUS method in post-EVAR follow-up. Although it does not constitute evidence validating use of CEUS for routine follow-up of EVAR, this study could be a first step to encourage further, more in-depth research into application of contrasted ultrasonographic examination for follow-up after endovascular repair of abdominal aortic aneurysms.

## CONCLUSIONS

CEUS is a technique that is easy to perform and which contributes additional diagnostic support for follow-up of endovascular repair of infrarenal abdominal aortic aneurysms.
