# Supplementary material for: Initial experience with Duplex scan combined with contrast-enhanced ultrasound for follow-up of endovascular abdominal aortic aneurysm repair
Source: J Vasc Bras. 2021 Nov 1;20:e20200093. doi: 10.1590/1677-5449.200093 (PMC9045535; doi:10.1590/1677-5449.200093)
Supplement: Translation

# Translation: Experiência inicial com ultrassom Doppler com contraste por microbolhas em adição ao ultrassom Doppler convencional para seguimento de correção endovascular de aneurisma de aorta abdominal

**Authors:** Carolina Brito Faustino, Carlos Ventura, Maria Fernanda Cassino Portugal, André Brunheroto, Marcelo Passos Teivelis, Nelson Wolosker

**Affiliations:** 1 Hospital Israelita Albert Einstein – HIAE, São Paulo, SP, Brasil.; 2 Faculdade de Medicina da Universidade de São Paulo – FMUSP, São Paulo, SP, Brasil.

**Keywords:** microbolhas, EVAR, endofuga, ultrassom

## Abstract

**Contexto:**

O ultrassom contrastado por microbolhas (CMUS) é uma modalidade diagnóstica de acurácia bem demonstrada por estudos internacionais para seguimento de reparo endovascular do aneurisma de aorta abdominal (EVAR). Não existem, no entanto, estudos nacionais focados nesse método de seguimento.

**Objetivos:**

O objetivo deste estudo foi relatar a experiência inicial com CMUS em um hospital terciário, traçando uma comparação dos achados do CMUS com o ultrassom Doppler convencional (USGD), com o intuito de verificar se a adição de contraste ao protocolo padrão de controle ultrassonográfico incorreu alteração nos achados.

**Métodos:**

Entre 2015 e 2017, 21 pacientes em seguimento de EVAR foram submetidos ao USGD seguido de CMUS. Foram avaliados os achados de exame referentes à identificação de complicações, bem como à capacidade de identificação da origem da endofuga.

**Resultados:**

Entre os 21 casos avaliados, 10 complicações foram evidenciadas no total: sete pacientes apresentaram endofuga (33,3%); dois pacientes apresentaram estenose em ramo de endoprótese (9,52%); e um paciente apresentou dissecção em artéria ilíaca externa (4,76%). Em 21 pacientes avaliados, o uso combinado dos métodos identificou 10 casos de complicações pós-EVAR. Em seis dos sete casos de endofugas (85,71%), o uso dos métodos combinados foi capaz de identificar a origem. O USGD isolado falhou na identificação da endofuga em dois casos (28,5%), identificando achados duvidosos em outros dois casos (28,5%), que obtiveram definição diagnóstica após associação do CMUS.

**Conclusões:**

O CMUS é uma técnica de fácil execução, a qual adiciona subsídios ao seguimento de EVAR infrarrenal.

## INTRODUÇÃO

Pacientes em pós-operatório de tratamento com reparo endovascular do aneurisma da aorta abdominal (EVAR) necessitam de seguimento virtualmente perene ao longo da vida para vigilância de possíveis complicações relacionadas ao procedimento, como crescimento aneurismático persistente, nova formação de aneurisma, ruptura potencial, vazamentos internos, entre outras^1^, sendo comumente recomendado seguimento em 1, 6 e 12 meses, seguido de exame anual^2^. A angiotomografia computadorizada (ATC) é atualmente a modalidade de imagem de escolha para seguimento pós-operatório^2^^,^^3^. Entretanto, existem preocupações em relação ao custo e a riscos de efeitos clínicos colaterais^4^^-^6.

A ultrassonografia com Doppler (USGD) surgiu como uma alternativa não invasiva, acurada e menos dispendiosa. Essa modalidade de avaliação, no entanto, apresenta limitações inerentes ao método, como nos casos de pacientes obesos, com meteorismo abdominal e com ateromatose extensa da parede arterial. Além disso, trata-se de um método que depende do operador, em que a variabilidade inter e intra-observador pode comprometer resultados consistentes^7^. Com a agregação de ferramentas tecnológicas ao USGD, como a técnica de fusão de imagens e volumetria, o exame tornou-se ainda mais sensível e específico^8^^-^10, proporcionando maior acurácia diagnóstica para eventuais complicações, a ponto de ser sugerido, por alguns autores, como possibilidade menos invasiva para substituição da angiotomografia no seguimento do EVAR^11^.

O uso de contraste ultrassonográfico (CMUS) surgiu como uma técnica adicional^12^^,^^13^, proposta por estudos internacionais como um método acurado para o seguimento de complicações após EVAR, com indicações específicas para verificação de endofuga e, em outros casos, estudo de morfologia de placas ateroscleróticas^1^^,^^7^^,^^14^^,^^15^. No entanto, não há estudos nacionais que abordem esse método de seguimento em nosso meio.

O objetivo deste estudo foi relatar a experiência inicial com CMUS em um hospital terciário da cidade de São Paulo, no estado de São Paulo (SP), traçando uma comparação dos achados dos métodos com e sem contraste para verificar se a adição de contraste ao protocolo padrão de USGD incorreu alguma alteração nos achados.

## MÉTODOS

Foram avaliados 21 pacientes realizando seguimento pós-operatório de EVAR entre 2015 e 2017 no Hospital Israelita Albert Einstein, São Paulo, SP, Brasil. Todos pacientes eram do sexo masculino, com idades entre 65 e 83 anos (média 80 anos, mediana 73 anos), para quem foi solicitado USGD como método de seguimento.

O estudo foi aprovado pelo Comitê de Ética da instituição para estudos envolvendo seres humanos, sob o número de protocolo 06201218.1.0000.0071 (parecer número 3.745.321).

Para esses pacientes, a solicitação da ultrassonografia foi devida, em oito casos, à necessidade de substituição da ATC por outro método devido a contraindicação (função renal limítrofe). Em um caso, o exame foi solicitado como complementar para tentativa de esclarecimento de achado em ATC sugestivo de endofuga, porém não foi completamente esclarecido. Nos 12 casos restantes, o exame ultrassonográfico foi solicitado como primeira escolha naquele momento do seguimento.

Os pacientes foram submetidos ao exame de USGD convencional e, em seguida, foi realizada a técnica com adição do contraste de microbolhas (CMUS) (Figura 1). Os achados obtidos após combinação dos métodos foram considerados referência para comparação entre uso do USGD isolado *versus* USGD associado ao CMUS.

**Figura 1:** Saco aneurismático de aorta abdominal com endoprótese bifurcada sem alterações. (A) Corte transversal; (B) corte longitudinal. Ambas as imagens mostram as duas pernas da endoprótese pérvias e com morfologia habitual (setas contínuas), sem evidência de vazamentos. As duas figuras mostram um recurso do aparelho que apresenta a imagem dividida em duas (*side by side*); imagem à esquerda com *preset* de contraste (harmônica invertida) e à direita mostrando imagem modo-B para comparação.

Em nossa instituição, o custo de adição de contraste ao exame de USGD foi de 30,4% (R$ 516,03) por exame de aorta abdominal.

### Técnica de exame

Os exames foram realizados preferencialmente na parte da manhã, após jejum de 6 horas. Foram utilizados os aparelhos EPIQ e IU 22 (Philips, Montana, EUA) e transdutor convexo (entre 1-5 MHz e de 2-5 MHz), com parâmetros de processamento otimizados.

Todos os exames foram realizados e laudados por um único examinador. Os pacientes foram avaliados em decúbito dorsal e eventualmente em decúbito lateral esquerdo e/ou lateral direito, com avaliação inicial em modo B e subsequente adição do modo cor, seguido pelo uso do contraste em *preset* adequado e terminando com reavaliação no modo cor para verificação de achados, conforme protocolo detalhado a seguir.

### Exame no modo B

O exame no modo B, que incluiu avaliação longitudinal e transversal da aorta e saco aneurismático, teve como objetivos principais a avaliação da morfologia da aorta, da posição e integridade da endoprótese e das dimensões e ecogenicidade do saco aneurismático (áreas anecoicas foram consideradas sugestivas de local de vazamento).

### Exame no modo cor

Os parâmetros de processamento de sinal do aparelho foram regulados com uso de ganho máximo e menor escala possível para formação de imagem adequada, intencionando máxima sensibilidade na detecção de fluxo para confirmação de vazamentos.

### Exame com adição de contraste (CMUS)

Foi utilizado o contraste Sonovue, comercializado na forma de um *kit* contendo hexafluoreto de enxofre em pó e uma seringa de solvente (5 mL). O manuseio durante preparo e injeção deve ser lento para evitar ruptura das microbolhas.

Um *bolus* de contraste de 1,0 mL seguido de um *flush* de 10 mL de solução salina foi administrado ao paciente por injeção endovenosa lenta, utilizando-se preferencialmente acesso em veia antecubital, com uso de uma agulha 18 G. Foi utilizado *preset* específico para uso de contraste disponível no aparelho, regulado com harmônica de pulso invertido e com baixo índice mecânico (0,4). Outra adequação importante para aplicação do uso de contraste é a retirada da zona de foco do aparelho da área estudada para evitar ruptura precoce das microbolhas. O procedimento de injeção de contraste foi repetido até três vezes quando necessário.

### Finalização do exame

Após o exame contrastatado, ele foi refeito em Doppler colorido para verificação de achados. É importante notar que o ganho do Doppler colorido deve ser ajustado para evitar artefato de espalhamento causado pela presença de contraste residual.

A análise do comportamento dinâmico das microbolhas foi realizada tanto por imagens estáticas quanto contínuas, em diversas fases da circulação do contraste. Foram registradas as sequências de imagens dinâmicas no modo cine por um *buffer* de quadro digital para posterior análise.

O temporizador foi ativado desde o início de cada injeção para analisar notadamente aqueles realces/vazamentos mais tardios e consequentemente de mais baixa intensidade.

### Análise de dados e avaliação estastítica

Foram analisadas as indicações dos exames, a ocorrência de complicações e a capacidade de diagnóstico das lesões. Nos casos específicos de ocorrência de endofugas, foi avaliada também a diferença de capacidade dos métodos para detecção da origem do vazamento. Os métodos de USGD e CMUS foram comparados quanto à capacidade de detecção de vazamento no interior saco aneurismático.

## RESULTADOS

A solicitação do exame para todos os casos veio dos cirurgiões vasculares responsáveis pelos casos, com intenção de seguimento pós-EVAR e avaliação de possíveis complicações. Não houve complicações relacionadas à realização do exame em nenhum dos métodos.

Em 21 pacientes avaliados, houve diagnóstico de 10 casos de complicações pós-EVAR (47,62%) (Tabela 1). Entre os casos de endofugas (33,33%), quatro foram classificadas pelo CMUS como tipo II, duas, como tipo Ib e uma, como tipo III.

**Tabela 1:** Complicações identificadas pós-EVAR no grupo de pacientes submetidos aos métodos combinados.

	**Identificados à USGD isolada, n (%)**	**Identificados à USGD com adição de CMUS, n (%)**
Endofugas*	3 (14,28)†	7 (33,33)
Tipo II	4 (57,14)	4 (57,14)
Tipo Ib	2 (28,57)	2 (28,57)
Tipo III	0	1 (14,28)
Adequada identificação da origem	6 (85,71)‡	7 (100)
Estenoses de ramo de endoprótese	2 (9,52)	2 (9,52)
Dissecção ilíaca	1 (4,76)	1 (4,76)

* Todas as endofugas identificadas foram do tipo II;

† Em dois casos, a USGD identificou alterações sugestivas de endofuga, porém sem definir o diagnóstico;

‡ Após determinação da existência das endofugas e classificação de sua origem pelo CMUS, a USGD isolada foi capaz de identificar a origem do vazamento em 6 dos 7 casos. EVAR = reparo endovascular do aneurisma da aorta abdominal; USGD = ultrassonografia com Doppler.

### Comparação dos métodos

Em relação à capacidade de detecção de vazamento no interior do saco aneurismático, dos 7 casos de vazamento identificados após adição do CMUS, dois não foram detectáveis à USGD isolada (28,5%) (Figura 2). Em dois outros casos (28,5%), a USGD identificou achados que poderiam ser considerados vazamentos, porém foram mais bem caracterizados, com confirmação do diagnóstico de endofuga, após associação do CMUS (Figuras 3-5).

**Figura 2:** Saco aneurismático de aorta abdominal com endoprótese bifurcada e com vazamento discreto e tardio. (A) Ultrassonografia com Doppler colorido com corte transversal mostra as duas pernas da endoprótese (setas contínuas) e ausência de vazamento; (B) Contraste ultrassonográfico mostra que existe vazamento discreto à esquerda do saco aneurismático (setas tracejadas). Ainda, a imagem é dividida em duas (*side by side*), recurso do aparelho já referido na Figura 1.

**Figura 3:** Saco aneurismático de aorta abdominal com endoprótese bifurcada e com vazamento tipo IB. (A) Ultrassonografia Doppler colorido no corte transversal mostra as duas pernas da endoprótese (setas contínuas) e pequeno vazamento junto a porção distal da perna esquerda da endoprótese (seta tracejada), que no exame dinâmico confirmou ser vazamento tipo IB; (B) Contraste ultrassonográfico mostra que o vazamento tem extensão bem maior do que parecia ao Doppler colorido (setas tracejadas). Ainda, a imagem é dividida em duas (*side by side*), recurso do aparelho já referido na Figura 1.

**Figura 4:** Saco aneurismático de aorta abdominal, com endoprótese bifurcada e com vazamento do tipo II por artéria lombar direita. (A) Ultrassonografia Doppler de amplitude no corte transversal mostra as duas pernas da endoprótese pérvias (setas contínuas) e ausência de vazamentos; (B) Contraste ultrassonográfico no corte transversal consegue evidenciar vazamento na porção posterior direita do saco aneurismático (seta tracejada) proveniente de artéria lombar (pontas de setas). Ainda, a imagem é dividida em duas (*side by side*), recurso do aparelho já referido na Figura 1.

**Figura 5:** Saco aneurismático de aorta abdominal com endoprótese bifurcada e com vazamento tipo III. (A) Ultrassonografia com Doppler colorido no corte transversal mostra o corpo da endoprótese à esquerda pérvio e com fluxo (seta continua); (B) Contraste ultrassonográfico no corte transversal mostra que o vazamento é mais importante (setas tracejadas) além de caracterizar que o vazamento é do tipo III, em contato com o corpo da prótese (seta tracejada). Ainda, a imagem é dividida em duas (*side by side*), recurso do aparelho já referido na Figura 1.

Relativo à capacidade de detecção da origem de endofugas, após determinação da existência das endofugas e classificação de sua origem pelo CMUS, o USGD isolado foi capaz de identificar a origem do vazamento em seis dos sete casos (85,71%).

## DISCUSSÃO

A adição do CMUS ao exame de Doppler convencional possibilita a avaliação vascular estrutural da macrocirculação (presença ou ausência de fluxo no vaso e morfologia da luz) e da microcirculação e a avaliação da contrastação ou perfusão tecidual (caracteriza o padrão e a intensidade de captação tecidual)^12^. O método contrastado fornece informações detalhadas do suprimento sanguíneo nas lesões teciduais, similares àquelas fornecidas pela ATC e pela angiorressonância magnética (ARM), por incluir as informações hemodinâmicas nas diferentes fases vasculares; com isso, encontrou aplicação inicialmente na avaliação de nódulos hepáticos, com boa acurácia^16^^,^^17^, sendo, eventualmente, expandido seu uso para diversas áreas.

Para a avaliação da aorta e dos vasos periféricos, diversos estudos corroboram a aplicação desse método, em especial no tocante ao acompanhamento após EVAR, para pesquisa de endofugas e de outras complicações^7^^,^^11^^,^^14^^,^^18^^-^22. O CMUS já foi também estudado na análise de doenças carotídeas, notadamente no diagnóstico diferencial entre oclusão e pseudo-oclusão da artéria carótida interna e na caracterização de microcirculação nas placas instáveis de carótidas^20^^,^^21^^,^^23^^,^^24^.

Atualmente, os meios de contraste ultrassonográfico mais difundidos são os gases fluorcarbonados, de alto peso molecular. Isso determina menor solubilidade e difusibilidade, proporcionando maior estabilidade às microbolhas, que persistem na circulação de forma prolongada (por até 10 min), garantindo boa sensibilidade ao método^25^^-^27. Por apresentarem diâmetros variando de 1 a 10 μm^19^, próximos àqueles das hemácias (7 μm), as microbolhas mimetizam seu comportamento, permitindo adequada avaliação mesmo na microcirculação^12^^,^^13^. A interação com a albumina humana, fosfolipídeos, surfactantes e outros compostos^28^ age estabilizando as microbolhas, reduzindo sua difusão, não só contribuindo com sua permanência na circulação, mas também aumentando a capacidade das bolhas de suportar variações de pressão decorrentes dos efeitos do ultrassom e da contração cardíaca^25^. O uso de contraste ultrassonográfico é contraindicado em pacientes com *shunts* cardíacos, hipertensão pulmonar ou condições cardiopulmonares instáveis (infarto agudo do miocárdio, síndromes coronarianas agudas, insuficiência cardíaca congestiva instável, arritmias ventriculares graves ou insuficiência respiratória, incluindo os pacientes que receberam ventilação mecânica), assim como pacientes com hipersensibilidade a perfluorcarbono^29^.

Em nossa amostra, não houve casos de reações adversas ao uso de contraste. Os efeitos adversos mais frequentemente relatados em literatura são dor ou parestesia no local da punção, dor lombar e, raramente, reações de alergia e danos à microcirculação^30^. Raras reações graves foram relatadas, como parada cardíaca ou respiratória fatal, perda de consciência, convulsões, arritmias sintomáticas (fibrilação atrial, taquicardia supraventricular, taquicardia ou fibrilação ventricular), hipotensão arterial, desconforto respiratório ou isquemia cardíaca^31^. É recomendável observar de perto os pacientes durante e após a administração do meio de contraste, bem como ter uma equipe de ressuscitação cardiopulmonar e equipamentos disponíveis.

Uma revisão recente de 18.942 estudos consecutivos em ecocardiografia evidenciou que a taxa de mortalidade a curto prazo (24 horas) é similar entre pacientes que receberam o meio de contraste sônico (Definity^®^, Bristol-Myers

Squibb Medical Imaging, Inc., Nova Iorque, EUA) durante exames de ecocardiograma em repouso e pacientes que não receberam o contraste, sem diferença de mortalidade^32^. Um estudo multicêntrico prospectivo, publicado em 2008 no *The American Journal of Cardiology* 13, também não demonstrou aumento da mortalidade em pacientes submetidos à ecocardiografia com contraste, quando comparados a pacientes submetidos a exames sem contraste. Esses resultados indicam que não há nenhum risco adicional associado a estudos realizados com CMUS.

Para traçar uma comparação entre o CMUS e outros métodos de imagem disponíveis – angiografia com subtração digital (ASD), ATC, ARM e mesmo a arteriografia –, é importante considerar o potencial nocivo do contraste iodado e do gadolíneo^4^^,^^5^^,^^33^. O contraste iodado, usado tanto na ASD como na ATC, pode apresentar reações adversas imediatas, que ocorrem em 3,1% dos pacientes, quando utilizado contraste de baixa osmolaridade^4^^,^^5^^,^^34^. O quelato de gadolínio, meio de contraste usado na ressonância magnética, também é uma fonte de reações adversas^6^^,^^35^. A maior parte dessas reações são leves e podem ser tratadas no próprio Departamento de Radiologia^4^. Deve-se considerar também que muitos pacientes com história de reação ao meio de contraste iodado parecem ter um risco aumentado de desenvolver alergia ao quelato de gadolínio^4^^,^^5^.

Em nosso estudo, identificamos que a adição de contraste ao protocolo de seguimento ultrassonográfico de EVAR elevou a capacidade de detecção de endofugas em comparação à utilização isolada de USGD. O método combinado elevou também, na nossa amostra, o número de acertos na identificação da origem das endofugas. Em concordância, a revisão do *Cochrane Vascular Group* publicada em 2017^7^, que analisou a acurácia diagnóstica da USGD e do CMUS em termos de sensibilidade e especificidade para detecção de vazamento interno após EVAR, demonstrou que ambas as modalidades de USG (com ou sem contraste) apresentaram alta especificidade. Para diagnosticar vazamentos, no entanto, os autores concluíram que o CMUS demonstrou ser superior à USGD. Os autores concluíram também que, em um programa de vigilância de endofugas, o CMUS pode ser introduzido como uma modalidade diagnóstica de rotina, seguida por ATC somente quando o ultrassom positivo estabelecer o tipo de vazamento interno e o tratamento terapêutico^7^.

Outra revisão sistemática de vigilância após EVAR, realizada por Zaiem et al. e publicada em 2018 no *Journal of Vascular Surgery* 1, avaliou a modalidade ideal e a frequência de vigilância após o EVAR em pacientes adultos com aneurismas da aorta abdominal. Nessa revisão, que identificou altas taxas de complicações, particularmente no primeiro ano, a ARM apresentou maior taxa de detecção de complicações entre os métodos, seguida pela tomografia computadorizada (TC). A USGD isolada mostrou-se menos sensível, porém mais específica que a TC para detecção de endofugas, ao passo que o CMUS teve alta sensibilidade. Nesse estudo, os resultados do método CMUS para detecção de vazamentos sugeriram equiparar-se àqueles demonstrada pela TC, com maior sensibilidade para identificação de endofugas tardias do tipo II. Tanto a USGD isolada quanto o CMUS foram altamente específicos para vazamentos do tipo I e III, e as estimativas de sensibilidade tenderam às mesmas taxas, porém com menor confiabilidade. Os autores postularam que as taxas mais altas de detecção de vazamento para o saco aneurismático advieram de abordagens de vigilância utilizando testes combinados; porém, a qualidade da evidência relativa ao diagnóstico nessa revisão foi moderada, pois adveio de estudos observacionais^1^.

A ATC é, até o momento, a técnica de escolha para seguimento de complicações após EVAR^36^ devido à sua alta sensibilidade e especificidade para avaliação de complicações, em especial de endofugas. Alguns autores sugerem, no entanto, que a ultrassonografia possa ser mais precisa na classificação do vazamento^15^, passo fundamental para definição de conduta. A ATC é capaz de avaliar vazamentos, mesmo que de baixo fluxo^37^; no entanto, não fornece informações sobre a direção do fluxo sanguíneo e, por isso, nem sempre consegue determinar corretamente a origem da endofuga ou classificá-la^38^. Nesse contexto, a ultrassonografia pode representar uma alternativa decisiva. Em consonância, em nossa amostra, a associação do CMUS à USGD determinou identificação da origem da endofuga em 85,7% dos casos.

Em um estudo de 2003, Bendick et al.^14^ avaliaram 20 pacientes em seguimento após EVAR, com ATC. Na amostra dos autores, oito casos de endofugas foram identificados pela ATC e igualmente identificados com CMUS. Em outros dois pacientes, o CMUS identificou endofuga tipo proximal, não diagnosticada pela ATC, mas confirmada por arteriografia (os autores supuseram que artefatos metálicos provavelmente prejudicaram a visualização do vazamento à ATC). Além disso, em três casos em que a classificação da endofuga não foi possível à ATC, o método CMUS identificou corretamente o padrão^14^.

Napoli et al.^39^, em seguimento de 112 pacientes submetidos a EVAR e seguidos com ATC, avaliaram o papel do CMUS em um grupo de pacientes diagnosticados com crescimento do saco aneurismático, sem identificação de vazamento por ATC ou USGD. Os pacientes foram divididos em três grupos: pacientes com crescimento do saco aneurismático, sem identificação de vazamento na ATC e USGD (grupo A); pacientes sem crescimento do saco aneurismático e sem vazamento diagnosticado (grupo B); e pacientes com vazamentos diagnosticados e classificados pela ATC (grupo C). Os pacientes do grupo A e C foram ainda submetidos a arteriografia para confirmação. O CMUS corretamente identificou presença de vazamento em todos os pacientes do grupo A, e a classificação do tipo em 80% deles. A arteriografia confirmou o resultado em 8 de 10 pacientes. A arteriografia também não foi capaz de esclarecer a dúvida nos casos em que a classificação do tipo de endofuga não foi feita pelo CMUS. Nos grupos controle, o CMUS não apresentou falsos positivos ou falsos negativos^39^.

Em estudo publicado em 2006^40^, 10 pacientes com endofuga diagnosticada à ATC foram submetidos ao CMUS para classificação ou reclassificação do vazamento. O CMUS confirmou a classificação da ATC em sete casos. Houve três casos em que a classificação da endofuga divergiu entre os métodos: nos três casos conflitantes, a arteriografia confirmou a classificação conforme determinada pelo CMUS. Os autores deste estudo postularam que o CMUS permite uma melhor avaliação da origem do vazamento, pois mostra o fluxo em tempo real e é mais específico que a ATC na classificação da endofuga, fornecendo informações mais precisas para o planejamento terapêutico^40^.

Embora a ATC mantenha-se um método de seguimento indispensável e considerado padrão ouro no pós-operatório de EVAR, devido à capacidade de avaliação mais precisa da ancoragem, integridade e morfologia da endoprótese^3^, estudos sugerem que o método CMUS é seguro e não-inferior à ATC no diagnóstico de complicações, podendo ser utilizado como seguimento padrão, com eventuais complementos pela ATC conforme necessário^7^. Alguns autores propõem ainda que esse método seja mais confiável que a ATC na classificação e avaliação do vazamento. O método tem indicação, ainda, em casos de crescimento de saco aneurismático, sem achado de vazamento pela ATC e no seguimento de endofugas tipo II, com redução de custos e de exposição a contraste iodado e radiação^40^.

Embora a adição do CMUS ao protocolo convencional de ultrassonografia para seguimento do EVAR tenha, neste e em outros estudos, demonstrado tratar-se de uma ferramenta com capacidade de alterar positivamente os resultados da investigação diagnóstica, até a conclusão deste estudo não há evidência que corrobore seu uso rotineiro ou em substituição a outros métodos, bem como não há ainda estudos de custo-benefício no território nacional.

## LIMITAÇÕES

Este é um estudo baseado na experiência inicial de uma instituição com aplicação do método contrastado ao seguimento ultrassonográfico, com amostra limitada, e cujo objetivo foi limitado na observação de achados adicionais que o uso do contraste trouxe ao protocolo convencional do exame de USGD para seguimento pós-EVAR. O estudo foi baseado em amostra heterogênea, pois inclui casos em que havia contraindicação à ATC, assim como casos previamente submetidos à ACT com limitações.

A mais importante limitação do estudo é não traçar uma comparação dos métodos (USGD isolada e USGD associada ao CMUS) com a angiotomografia, atualmente considerada padrão ouro no seguimento pós-EVAR^2^^,^^3^. Uma vez que o parâmetro utilizado foram os achados do método combinado, este estudo dispõe-se apenas à verificação do benefício da adição do meio de contraste ao exame de USGD convencional, não tendo desenho que permita validação de método diagnóstico.

Estudos especificamente desenhados para validação de parâmetros diagnósticos são necessários para definição, em nosso meio, da aplicação ideal do método CMUS ao seguimento pós-EVAR. Embora não represente evidência para validação para o uso de CMUS no seguimento rotineiro do EVAR, este trabalho pode representar o passo inicial para o incentivo à pesquisa mais aprofundada referente à aplicação do exame ultrassonográfico contrastado no seguimento pós-correção endovascular de aneurismas da aorta abdominal.

## CONCLUSÃO

O CMUS é uma técnica de fácil execução que adiciona subsídio diagnóstico ao seguimento de correção endovascular de aneurismas de aorta abdominal infrarrenal.
